# Mal de Debarquement Syndrome: A Rare Entity

**DOI:** 10.7759/cureus.6837

**Published:** 2020-02-01

**Authors:** Shehzeen F Memon, Anosh Aslam Khan, Osama Mohiuddin, Shahzeb Ali Memon

**Affiliations:** 1 Internal Medicine, Dow University of Health Sciences, Karachi, PAK

**Keywords:** mal de debarquement syndrome, rare disease, benzodiazepines, dizziness, cruise, vertigo, persistent motion

## Abstract

Mal de debarquement syndrome (MdDS) is a bizarre sensation of continued movement after the termination of motion. It is accompanied by disequilibrium, usually experienced after voyage or travel, however, it is not associated with vertigo. Although most cases resolve spontaneously, middle-aged women sometimes particularly experience protracted symptoms following an ocean cruise, with the persistence of symptoms for many years. We present the case of a young female with no known comorbidities who was misdiagnosed quite a few times before the actual diagnosis of this rare disease was established.

## Introduction

Mal de debarquement syndrome (MdDS) is a one-of-its-kind illness characterized by unsteadiness without dizziness, which can persist for months or sometimes even years. The term Mal de Débarquement in French stands for "sickness of disembarkment". It is a diagnosis of exclusion, based on characteristic history and normal neurologic and otorhinolaryngology (ENT) clinical examination, however, nystagmus can also be observed [1]. It is a continuous sensation of rocking and swaying after a period of travel such as by ship, plane or car [2]. The symptoms often resolve spontaneously after a few days but in some cases, they can persist for a prolonged and unpredictable duration leaving a patient in the debilitated state with significant impairment in quality-of-life (QoL). Since the diagnosis remains clinical, testing is useful only in helping to exclude other disorders that might present with features similar to MdDS [3]. We present a case of a 20-year-old woman who came for the evaluation of frequent episodes of the persistent sensation of imbalance for the previous one and a half years. She was diagnosed as a case of MdDS based on typical history and relevant examination and investigations. She was started on low dose benzodiazepines which yielded relief from her symptoms. The patient was carefully followed up for six months and has been symptom-free thereafter.

## Case presentation

A 20-year-old female patient, with no-known co-morbidities presented to the neurology consult in March 2019 with the complaints of dizziness for one and half years and frequent episodes of gradually progressive, non-debilitating headaches for the last three months. The symptoms began two days following a turbulent 16-hour flight from Abu Dhabi to Los Angeles. Initially, mild dizziness persisted for a week. Subsequently, she started to feel a sensation of rocking and swaying where she felt as if her 'whole body was being pulled to the ground with a very strong force'. At times, she also felt like 'walking on a trampoline' and as if her 'brain was moving to and fro inside her head'. This sensation was constantly varying in intensity from day to day but never completely disappeared. However, she noticed that the intensity of the symptoms gradually increased which profoundly affected her functionality at home and college. Thereafter, it became difficult for her to concentrate on her upcoming exams. She visited neurosurgery consult where she was told that her symptoms might be due to exam stress as her neurological examination and magnetic resonance imaging (MRI) of the brain were all unremarkable. Nevertheless, her symptoms aggravated after exams. The patient often had to grab furniture and sidewalls for support because of a 'staggering' gait although she never fell. The rocking and swaying sensation were particularly worse while lying down at bedtime and seemed to be alleviated while riding or driving her car. She had no associated diplopia, nausea, vomiting, hearing deficits or tinnitus. She denied alcohol and illicit drug abuse. She also denied any history of head trauma. Her medical, surgical, family and social history were also insignificant. According to the patient, she never experienced similar symptoms in the past.

**Figure 1:**
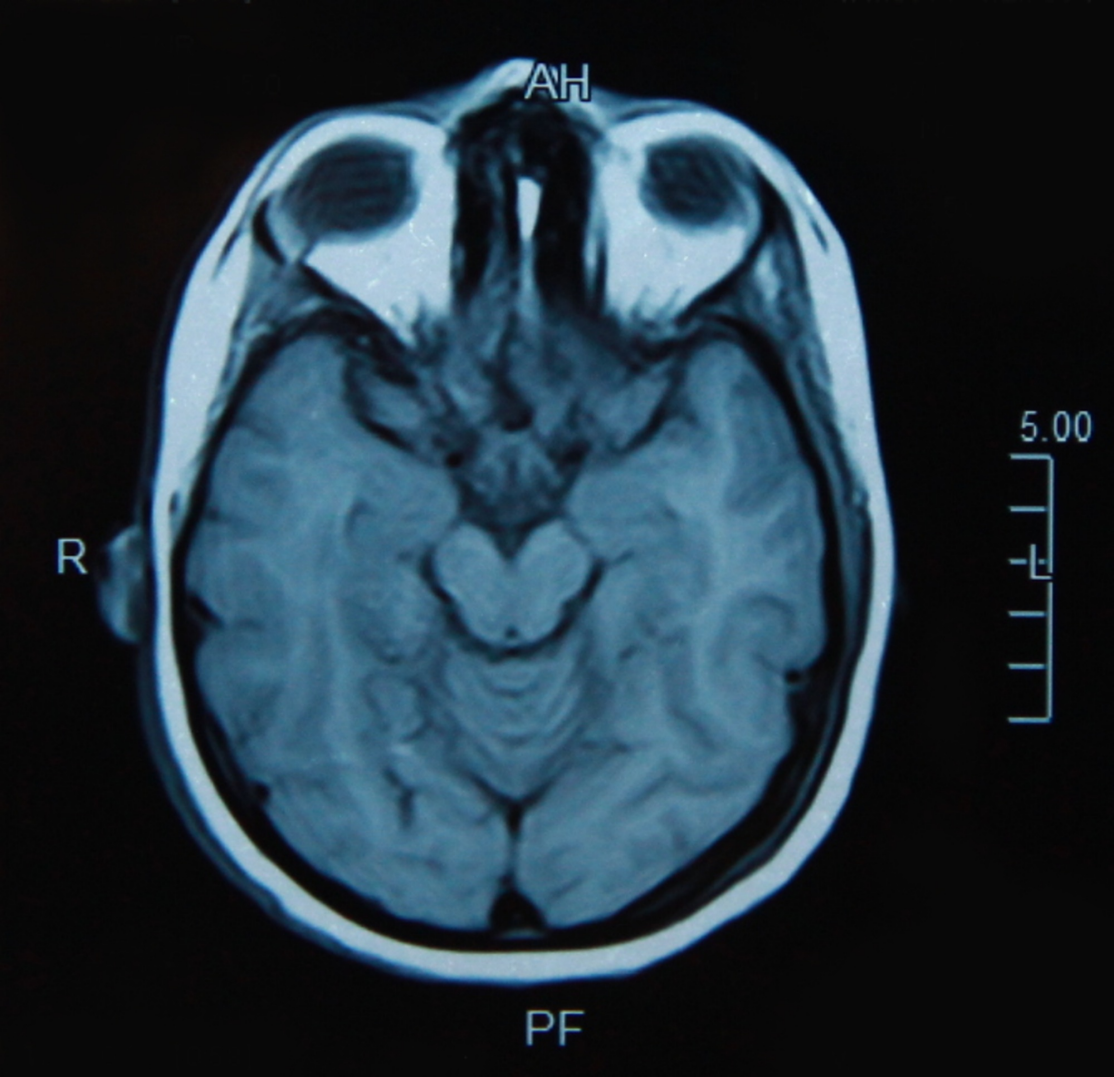
Magnetic resonance imaging (MRI) of the brain, T1-weighted image, axial view showing normal brain parenchyma without any radiological changes

**Figure 2:**
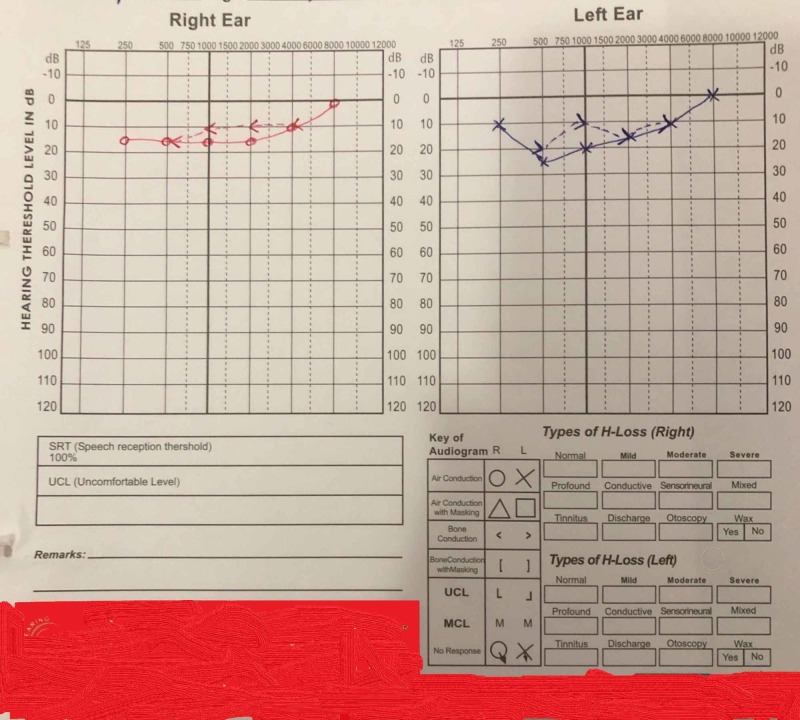
Pure-tone audiogram (PTA) showing normal air and bone conduction thresholds for the left and right ear

Prior to neurology consult, she also visited multiple physicians and otorhinolaryngologists who prescribed various motion sickness therapies including scopolamine and meclizine but none of them were responsive to her symptoms. On examination, the patient was fully responsive, alert and oriented with normal effect. Her vitals were within normal limits. Her head was normocephalic and atraumatic. A complete mental status exam was performed which was normal. Detailed neurological examination including cranial nerve examination, sensory examination, deep tendon reflexes and cerebellar function assessment (including hand coordination and past pointing) did not reveal any pathology. The balance test including tandem stance and Romberg sign also turned out to be normal. The patient's pupils were bilaterally equal, round and reactive to light and accommodation. Extra-ocular movements were normal and there was no abnormality on fundoscopic examination. Vestibulo-ocular function test was sought which showed normal saccadic and smooth pursuit of eye movements. There was no sign of spontaneous or gaze-holding nystagmus. Other relevant examinations consisting of head thrust, bilateral roll, Dix-Hallpike, and vestibulo-ocular reflex were also normal. An otorhinolaryngology (ENT) consult was obtained as the extensive neurological examination was unremarkable. However, no abnormality was observed on the otorhinolaryngological (ENT) examination. Subsequently, MRI of the brain and audiogram were sought as a final resort (Figures 1-2). Both of them revealed inconsequential results. Basic laboratory workup including complete blood count and serum electrolyte levels were also within a normal range.

In the setting of typical traveling history, normal laboratory tests and unremarkable neuro-otological examination, a provisional diagnosis of MdDS was made. She was initiated on low dose benzodiazepine (clonazepam 0.5 mg twice daily). She was also offered physical therapy which focused on vestibular/balance rehabilitation. Within a couple of weeks, the patient began to feel some improvement in her symptoms, which completely resolved within six weeks. The patient was followed up in the outpatient department for six months to keep a check on spontaneous recurrence. However, she remains symptom-free to date.

## Discussion

MdDS is not a well-defined pathological disease rather a sensation provoked by movement unassociated with abnormalities of cranial nerves or auditory system, as evident in our patient [4]. Cha et al. reported 64 patients around an average of 39 years of age while our patient was a young female in her 20s [5]. Literature suggests that patients with persistent MdDS have a high metabolic rate for glucose in the amygdala and the left entorhinal cortex (an area that processes spatial information) and decreased metabolism prefrontal and temporal cortices [4]. These changes are commonly observed using the F-fludeoxyglucose positron-emission tomography (F-PET) scan. In 2012, this scan was used for the first time on 20 patients in New York [6-7]. The subjects showed increased connectivity between the entorhinal cortex and sensory processing areas located in the parietal and occipital lobes (V1, V5, superior parietal lobule), whereas connectivity with the prefrontal and premotor cortices and homologous structures in the prefrontal cortex was reduced. In Pakistan, an F-PET scan is a highly expensive investigation not commonly performed and so our patient did not undergo any such tests. Kikkeri et al. reported a 47-year-old woman who was diagnosed as MdDS when she presented with the feeling of imbalance following a cruise, which temporarily subsides during a bicycle ride or a car drive [8]. She described the symptom as a feeling of constant motion throughout the day, which persisted while lying down. Her symptoms got better when motion stopped while our patient had a slight intensity of symptoms throughout even though her headache couldn't be classified to be migraine as she had no history of aura, photophobia, phonophobia, unilateral involvement or any known triggers for migraine attacks. Cha et al. also reported migraine as a common association to this syndrome [5]. Similar to our patient, this patient’s nystagmography (VNG), oculomotor testing, positional testing, and audiogram were all normal. This syndrome is further investigated by plain CT scan of the brain and temporal bone, and MRI screening for the brain, both of which are investigations for exclusion as they do not show any abnormal signals. All the above-mentioned radiological investigations were normal in our patient. 

There is no definite cure for this syndrome. However, it is managed by benzodiazepines, anti-emetics, selective serotonin reuptake inhibitors, tricyclic antidepressants, beta-blockers or anticonvulsants. Most patients try anti-emetics as the first-line treatment however in most cases it is not enough and an added treatment needs to be given [9]. Our patient received anti-emetics but her symptoms improved only on benzodiazepines. Some patients who do not benefit from pharmacological management also receive vestibular rehabilitation; theta burst stimulation on the occipital cortex and cerebellar hemisphere and vermis [9-10]. These tests are quite new to our healthcare system and will need more research to develop and be used as a routine procedure. Further studies need to be done on the subject of MdDS because this syndrome is an infrequent entity and clinical cases seldom sprout and thus, scarce literature is available on the subject, especially in South Asia. Physicians, in this part of the world, are generally unaware of this condition and as a result, patients are often misdiagnosed and some patients may never receive the diagnosis and a customized management plan.

## Conclusions

MdDS is an unusual and understudied disorder with a high index of suspicion for its diagnosis. Due to its rare occurrence, fairly in-depth studies are required to understand its pathophysiology, symptomatology, risk factors, and diagnostic and curative measures so that efforts can be made to bring the lives of such patients towards normality.
